# Upregulation of *FAM83D* affects the proliferation and invasion of hepatocellular carcinoma

**DOI:** 10.18632/oncotarget.4432

**Published:** 2015-06-10

**Authors:** Weijia Liao, Weilong Liu, Xing Liu, Qing Yuan, Ying Ou, Yao Qi, Wanqiu Huang, Yun Wang, Jian Huang

**Affiliations:** ^1^ Laboratory of Hepatobiliary and Pancreatic Surgery, Affiliated Hospital of Guilin Medical University, Shanghai Center for Systems Biomedicine, Shanghai Jiao Tong University, Shanghai, China; ^2^ Key Laboratory of Systems Biomedicine (Ministry of Education), Collaborative Innovation Center of Systems Biomedicine, Shanghai Center for Systems Biomedicine, Shanghai Jiao Tong University, Shanghai, China; ^3^ Shenzhen Key Laboratory of Infection and Immunity, Shenzhen Third People's Hospital, Guangdong Medical College, Shenzhen, China; ^4^ Shanghai-MOST Key Laboratory for Disease and Health Genomics, Chinese National Human Genome Center, Shanghai, China; ^5^ Tongji Medical College, Huazhong University of Science and Technology, Wuhan, China

**Keywords:** hepatocellular carcinoma, FAM83D, up-regulation, overall survival, methylation

## Abstract

The identification of potential oncogenes plays an important role in finding novel therapeutic targets for many cancers, including hepatocellular carcinoma (HCC), which is one of the most common cancers worldwide. In our previous research, using microarray technology, we found that *FAM83D* was overexpressed in HCCs. However, whether the overexpression of *FAM83D* contributes to hepatocarcinogenesis remains unclear. In this study, we found that *FAM83D* was significantly upregulated in 76.6% (167 of 218) of the HCC specimens at the mRNA level and in 69.44% (50 of 72) of the HCC specimens at the protein level compared with adjacent non-cancerous liver specimens, as indicated by RT-PCR and immunohistochemical staining, respectively. The *FAM83D*mRNA expression level was positively correlated with the level of alpha-fetoprotein (AFP) (≥100 ng/ml), the clinical TNM stage, the presence of a portal vein tumor thrombus (PVTT), disease-free survival (DFS) and the overall survival (OS) time of the HCC patients (*P* < 0.05). Knocking down *FAM83D* significantly promoted the growth of Huh7 and HepG2 cells, as demonstrated in an RNA interference assay. Moreover, the DNA methylation status of the *FAM83D* promoter was significantly reduced in the HCC specimens with overexpression of *FAM83D* gene. Our data suggest that the upregulation of *FAM83D*, a potential oncotarget gene, may be triggered by epigenetic events and can contribute to hepatocarcinogenesis.

## INTRODUCTION

Hepatocellular carcinoma (HCC), the sixth most prevalent type of cancer, is the third leading cause of cancer-related mortality worldwide [1]. The prognosis of HCC remains poor, mainly because the recurrence rates are high even after surgical resection; tumor recurrence complicates more than 70% of cases at five years after resection [2, 3]. Although surgical resection is a potentially curative treatment for HCC, and despite the availability of improved diagnostic techniques and advances in surgical and nonsurgical therapies for the disease, the clinical outcome of HCC remains poor [4]. Therefore, the identification of the molecular mechanisms underlying the development and progression of HCC is of particular importance. Such research might lead to a breakthrough in the field of HCC diagnosis, treatment and prevention, and it may enable improved post-surgical outcomes for HCC.

In this study, we report that that the expression of *FAM83D* is increased in HCC tissues, as assessed by an analysis of the whole-genome expression profiles on chips of hepatitis B virus-induced HCC. To further investigate the relationship between *FAM83D* and HCC in humans, we evaluated the expression level of *FAM83D* in HCC tissues using quantitative real-time PCR (qRT-PCR) and immunohistochemistry (IHC). Additionally, we conducted a comparative analysis with the clinical pathological data. Moreover, we compared and analyzed the expression levels of *FAM83D* in normal liver tissues, fetal liver tissues and HCC cell lines, and we examined its influence on the growth of HCC cell lines by silencing its expression using RNA interference (RNAi). This research provides a foundation for understanding the function of *FAM83D* in the development and progression of HCC.

## RESULTS

### *FAM83D* is upregulated at the mRNA level in HCC tissues

**Figure 1:**
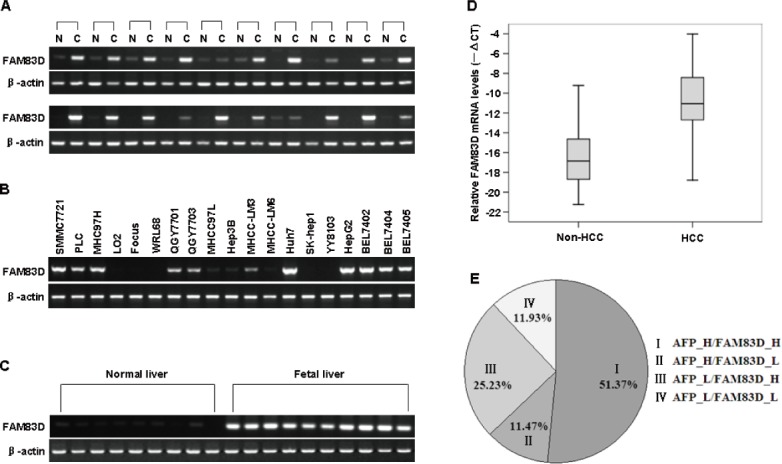
The results of RT-PCR and real-time RT-PCR analyses of the *FAM83D* mRNA expression in tissue specimens **A.**–**C.** The *FAM83D* mRNA level was measured in 20 HCC tissue specimens and in the corresponding adjacent non-cancerous (non-HCC) tissue samples **A.**, 19 human HCC cell lines **B.**, 9 normal liver tissue samples and 10 fetal liver tissue samples **C.** by RT-PCR. *ACTB* served as an internal control. The PCR products were visualized by electrophoresis on 2% agarose gels. **D.** The mRNA level of *FAM83D* in 218 paired HCC specimens and the adjacent non-cancerous liver tissue were determined by quantitative real-time PCR. *ACTB* was used as an internal control. The line within each box represents the median -ΔCt value; the upper and lower edges of each box represent the 75th and 25th percentiles, respectively; the upper and lower bars indicate the highest and lowest values determined, respectively. **E.** The results of a real-time RT-PCR analysis of the expression status of *FAM83D* and AFP in 218 HCC specimens examined. The numbers represent the percentages of *FAM83D*- and/or AFP-positive HCC specimens.

Using a semi-quantitative RT–PCR assay, we found that *FAM83D* was significantly upregulated in 90% (18/20) of the HCC specimens compared with the adjacent non-cancerous liver samples (Figure 1A). In addition, using the same technique, we found that *FAM83D* was highly expressed in the HCC cell lines SMMC7721, PLC, MHCC97H, QGY7701, QGY7703, Huh7, HepG2, BEL7402, BEL7404 and BEL7405, whereas there was weak expression in MHCC-LM6, MHCC-LM3, MHCC97L and Hep3B cells. There was no expression in the two normal liver cell lines LO2 and WRL68 or in the two HCC cell lines SK-hep1 and YY8103 (Figure 1B). Compared with normal liver tissues, *FAM83D* was more highly expressed in human fetal liver tissues (Figure 1C). This result implies that the expression level of *FAM83D* is closely related to the development of the liver. Considering the limitations of the RT-PCR method, the mRNA level of *FAM83D* was also evaluated in 218 cases by real time RT-PCR. Of these 218 cases, 76.6% (167) of the HCCs showed at least a 2-fold increase in the *FAM83D* mRNA level (normalized to the *ACTB* level) in each sample, compared with that of the corresponding non-cancerous livers. These data support the semi-quantitative RT-PCR results indicating that *FAM83D* is overexpressed in HCCs (*P* < 0.001, Figure 1D).

Interestingly, the expression of *FAM83D* and serum AFP did not completely overlap in the 218 HCC specimens examined by real-time RT-PCR. The expression of *FAM83D* and the serum AFP level were simultaneously high in 112 (51.37%) of the 218 HCC cases. *FAM83D* was not significantly expressed in only 25 (11.47%) of the 218 cases with a high AFP level. Surprisingly, overexpression of *FAM83D*, but not AFP, was evident in 25.23% (55 of 218) of the HCC specimens (Figure 1E), suggesting that FAM83D may be considered to be a novel candidate biomarker for the pathogenesis of HCC.

Notably, our current findings indicated that the upregulation of *FAM83D* was not significantly correlated with gender, age (≥50 or <50 years old), tumor size (≥5 or <5 cm), family history, HBsAg expression, the size and the number of tumors, the presence of liver cirrhosis, alcohol consumption, distant metastasis or lymph node metastasis, postoperative recurrence, the level of AST, or the NLR (*P* > 0.05, Table 1). However, the upregulation of *FAM83D* positively correlated (*P* < 0.05) with the level of AFP (*P* = 0.020), the presence of PVTT (*P* = 0.025) and the clinical TNM stage (*P* = 0.007) (Table 1).

**Table 1:** Correlation between the clinicopathologic variables and FAM83D expression in HCC

Clinical character	Clinical Groups	No.of patients	FAM83D		*x*^2^	*p* value
			High (%)	Low (%)		
Age (years)	< 55	142	107 (75.4)	35 (24.6)	0.357	0.550
	≥ 55	76	60 (78.9)	16 (21.1)		
Gender	Female	32	22 (68.8)	10 (31.2)	1.291	0.256
	Male	186	145 (78.0)	41 (22.0)		
Family history	No	183	142 (77.6)	41 (22.4)	0.624	0.430
	Yes	35	25 (71.4)	10 (28.6)		
HBsAg	Negative	39	31 (79.5)	8 (20.5)	0.220	0.639
	Positive	179	136 (76.0)	43 (24.0)		
AFP (ng/ml)	< 100	81	55 (67.9)	26 (32.1)	5.449	0.020
	≥ 100	137	112 (81.8)	25 (18.2)		
Tumor size (cm)	< 5	52	37 (71.1)	15 (28.9)	1.132	0.287
	≥ 5	166	130 (78.3)	36 (21.7)		
Cirrhosis	No	20	15 (75.0)	5 (25.0)	0.032	0.859
	Yes	198	152 (76.8)	46 (23.2)		
Tumor number	Single	146	111 (76.0)	35 (24.0)	0.082	0.774
	Multiple	72	56 (77.8)	16 (22.2)		
Wine-drinking	No	101	79 (78.2)	22 (21.8)	0.273	0.601
	Yes	117	88 (75.2)	29 (24.8)		
TNM stage	I–II	105	72 (68.6)	33 (31.4)	7.296	0.007
	III–IV	113	95 (84.1)	18 (15.9)		
PVTT	No	162	118 (72.8)	44 (27.2)	4.991	0.025
	Yes	56	49 (87.5)	7 (12.5)		
Metastasis	No	185	141 (76.2)	44 (23.8)	0.103	0.749
	Yes	33	26 (78.8)	7 (21.2)		
Recurrence	No	140	102 (72.9)	38 (27.1)	3.068	0.080
	Yes	78	65 (83.3)	13 (16.7)		
AST (U/l)	< 40	98	75 (76.5)	23 (23.5)	0.001	0.981
	≥ 40	120	92 (76.7)	28 (23.3)		
NLR	< 2.31	127	97 (76.4)	30 (23.6)	0.009	0.925
	≥ 2.31	91	70 (76.9)	21 (23.1)		

* FAM83D High: 2 − ΔΔCT > 1; FAM83D Low: 2 − ΔΔCT ≤ 1; HBsAg, hepatitis B surface antigen; AFP, alpha-fetoprotein; TNM, tumor-node-metastasis; PVTT, portal vein tumor thrombus; Metastasis, distant metastasis or lymph node metastasis; AST, aspartate aminotransferase; NLR, neutrophil to lymphocyte ratio.

**Figure 2:**
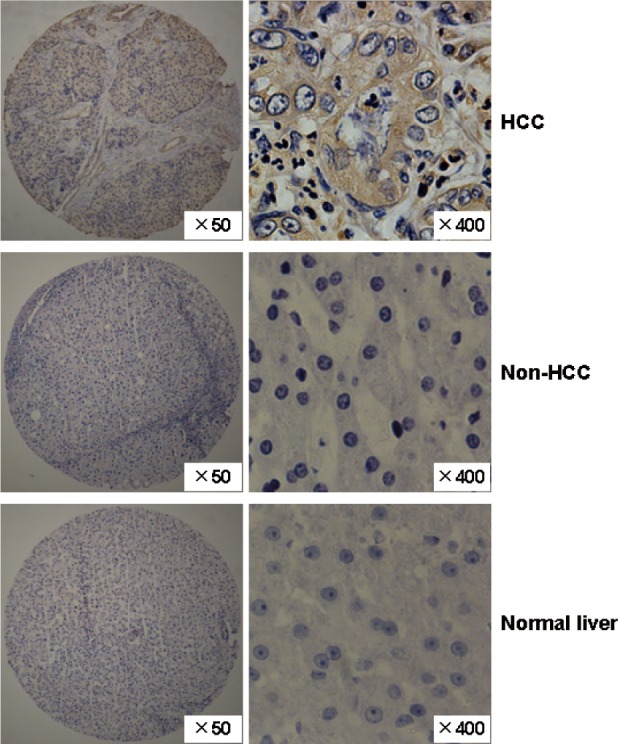
The expression pattern of the FAM83D protein in HCC specimens Representative immunohistochemical staining of a pair of HCC specimens and their corresponding non-cancerous tissues, and normal liver tissues, as determined using an anti-FAM83D antibody on a tissue array containing 72 pairs of HCC specimens. The nuclei were counterstained with hematoxylin. Original magnification, ×50 (left, tissue chip); ×400 (right; HCC, non-HCC and normal liver).

Furthermore, we used tissue chips to detect the expression of FAM83D at the protein level in 72 HCC tissues and the adjacent non-cancerous liver tissue specimens. We found that the FAM83D protein was overexpressed in 69.44% (50/72) of the HCCs, but only 12.5% (9 of 72) of the non-HCC tissues stained positively for FAM83D, and the difference in FAM83D staining between the HCC and non-HCC tissues was statistically significant (*P* < 0.001). The expression of the FAM83D protein was not detected in normal liver tissues (Figure 2).

### The relationship between *FAM83D* upregulation in HCCs and survival time

**Figure 3:**
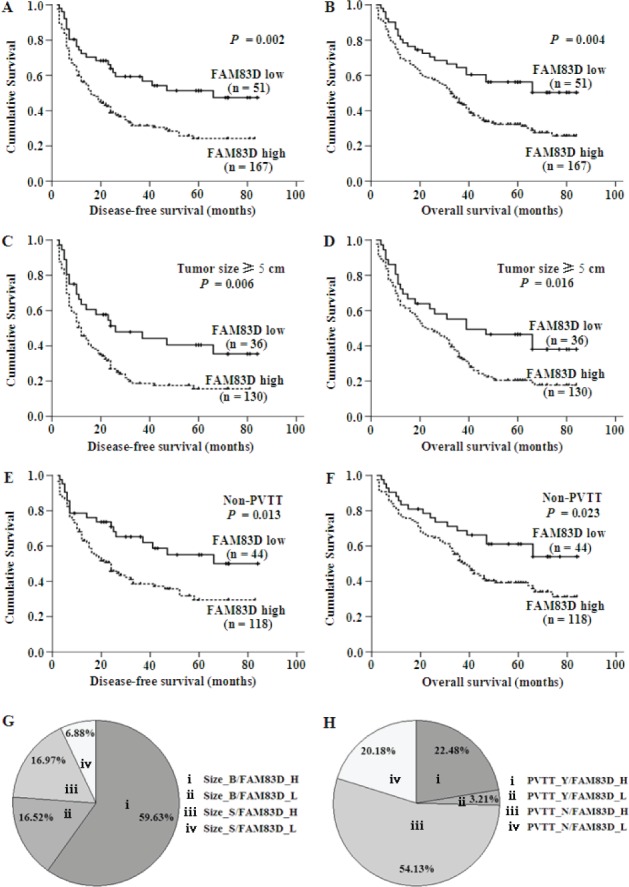
**A.–F.** The results of the Kaplan-Meier survival analysis showing that patients with high expression of *FAM83D* had a shorter DFS and OS. The green line represents the low expression of *FAM83D*, whereas the blue line represents the high expression of *FAM83D*. **G.** and **H.** The expression of *FAM83D* and a large tumor size or the presence of PVTT did not completely overlap in the 218 HCC specimens. *FAM83D* was overexpressed in 130 of the 218 HCC specimens (59.63%) with a tumor size ≥5 cm (Size_B), whereas *FAM83D* was not expressed in only 15 (6.88%) of the cases with a tumor size <5 cm (Size_S) (G). *FAM83D* was overexpressed in 118 of the 218 HCC specimens (54.13%) without PVTT (PVTT_N), moreover, *FAM83D* was not expressed in only 7 (3.21%) cases that had PVTT (PVTT_Y) (H).

A Kaplan-Meier survival analysis showed that a higher *FAM83D* expression level was associated with a shorter disease-free survival (DFS) and overall survival (OS) (Figure 3A and 3B). A univariate analysis revealed that there was an obvious association of the clinical parameters with both the DFS and OS (Table 2). The 5-year DFS rates of patients with low *FAM83D* expression was 51.8%, compared with 26.4% for patients with high *FAM83D* expression (*P* = 0.002; Figure 3A). The mean DFS in patients with low *FAM83D* expression was 50.61 months [95% confidence interval (*CI*), 40.81-60.44] compared with 32.15 months (95% *CI*, 27.04-37.12) in patients with high *FAM83D* expression. The 5-year OS rates of patients with low *FAM83D* expression was 57.5% compared with 32.6% for patients with high *FAM83D* expression (*P* = 0.004; Figure 3B). The mean OS values in the low and high *FAM83D* expression groups were 54.98 months (46.09-63.94) and 39.86 months (35.10-44.65), respectively. In addition to a high *FAM83D* expression level, a value of AFP ≥100 ng/ml, a tumor ≥5 cm, the presence of multiple tumors, TNM stage III-IV, the presence of PVTT, the presence of distant metastasis or lymph node metastasis, AST ≥40 U/l and NLR ≥2.31 were associated with a shorter DFS and OS (Table 2).

**Table 2:** Association between FAM83D expression level or clinical parameters and disease-free survival/overall survival

Clinical character	Category	No.of patients	Disease-free survival (months)			Overall survival (months)		
			Mean	95% CI	*p* value	Mean	95% CI	*p* value
FAM83D expression	Low	51	50.61	40.81–60.44	0.002	54.98	46.09–63.94	0.004
	High	167	32.15	27.04–37.12		39.86	35.10–44.65	
Age (years)	< 55	142	36.94	31.09–42.83	0.996	43.16	37.75–48.58	0.952
	≥ 55	76	36.25	28.72–43.68		44.16	37.09–51.22	
Gender	Female	32	38.81	27.66–49.89	0.212	52.03	40.88–63.19	0.144
	Male	186	35.53	30.60–40.53		41.99	37.36–46.64	
Family history	No	183	35.69	30.65–40.70	0.223	42.27	37.61–46.94	0.161
	Yes	35	44.42	32.32–56.54		51.02	39.90–62.13	
HBsAg	Negative	39	34.64	23.52–45.58	0.868	44.46	34.72–54.18	0.961
	Positive	179	37.08	31.91–42.24		43.28	38.47–48.07	
AFP (ng/ml)	< 100	81	44.43	37.27–51.59	0.002	51.86	45.59–58.14	0.001
	≥ 100	137	30.60	24.71–36.47		36.82	31.22–42.45	
Median size (cm)	< 5	52	63.62	55.10–72.08	< 0.001	68.88	62.23–75.55	< 0.001
	≥ 5	166	28.23	23.41–33.09		35.68	31.05–40.32	
Cirrhosis	No	20	30.79	15.61–46.04	0.379	38.11	24.35–51.86	0.493
	Yes	198	37.38	32.44–42.27		44.23	39.69–48.78	
Tumor number	Single	146	43.22	37.42–49.05	< 0.001	49.75	44.58–54.95	< 0.001
	Multiple	72	22.81	16.35–29.16		31.04	24.22–37.83	
Wine-drinking	No	101	41.14	33.84–48.49	0.155	47.27	40.59–5395	0.103
	Yes	117	33.76	27.66–39.78		40.62	35.02–46.22	
TNM stage	I-II	105	51.52	44.81–58.13	< 0.001	58.57	53.01–64.13	< 0.001
	III-IV	113	22.60	17.55–27.81		29.56	24.19–34.94	
PVTT	No	162	41.59	36.07–47.18	< 0.001	48.66	43.67–53.68	< 0.001
	Yes	56	22.47	15.37–29.42		28.86	21.63–36.10	
Metastasis	No	185	40.12	34.94–45.20	< 0.001	46.51	41.80–51.21	< 0.001
	Yes	33	17.33	10.13–24.52		26.78	18.14–35.42	
AST (U/l)	< 40	98	49.24	42.08–56.17	< 0.001	55.95	49.83–62.07	< 0.001
	≥ 40	120	27.03	21.26–32.64		33.65	28.26–39.08	
NLR	< 2.31	127	44.07	37.58–50.43	0.001	50.24	44.52–55.95	< 0.001
	≥ 2.31	91	26.93	20.82–33.09		34.39	28.29–40.51	
Recurrence	No	140				39.87	34.08–45.66	0.073
	Yes	78				48.67	42.40–54.94	

CI, confidence interval; HBsAg, hepatitis B surface antigen; AFP, alpha-fetoprotein; TNM, tumor-node-metastasis; PVTT, portal vein tumor thrombus; Metastasis, distant metastasis or lymph node metastasis; AST, aspartate aminotransferase; NLR, neutrophil to lymphocyte ratio.

While clarifying the specific subgroups of patients negatively influenced by *FAM83D* upregulation, we found that *FAM83D* was highly expressed in patients with tumors ≥5 cm and in the non-PVTT cases (Figure 3G, 3H), and the prognostic significance of *FAM83D* was retained in both of these subgroups (Figure 3C– 3F). In the subgroup with tumors ≥5 cm, high *FAM83D* expression showed apparent prognostic value for predicting a poorer DFS (*P* = 0.006) and OS (*P* = 0.016) (Figure 3C, 3D). In addition, the finding that high *FAM83D* expression significantly correlated with a shorter DFS (*P* = 0.013) and OS (*P* = 0.023) was observed in the subgroup of HCC with non-PVTT (Figure 3E, 3F). These findings suggest that the level of *FAM83D* may serve as a prognostic molecular marker for some subgroups of HCC patients.

After adjusting for other confounding factors, the 4 independent factors identified (high *FAM83D* expression in HCC tissues, AFP ≥100 ng/ml, tumor size ≥5 cm and AST ≥40 U/l) were used in a stepwise multivariate Cox proportional hazard model for both the DFS and OS, and the hazard ratio (HR), 95% confidence interval (CI), and *P* values of these four independent predictors are listed in Table 3. The analysis revealed that a value of AFP ≥100 ng/ml (HR, 1.515; 95% CI, 1.063-2.160, *P* = 0.021), tumor size ≥5 cm (HR, 2.768; 95% CI, 1.550-4.943, *P* = 0.001), AST ≥40 U/l (HR, 1.780; 95% CI, 1.226-2.584, *P* = 0.002), and high *FAM83D* expression (HR, 1.568; 95% CI, 1.112-2.209, *P* = 0.010) were all independent predictors for the DFS (Table 3). A value of AFP ≥100 ng/ml (HR, 1.770; 95% CI, 1.128-2.775, *P* = 0.013), tumor size ≥ 5 cm (HR, 2.622; 95% CI, 1.470-4.678, *P* = 0.001), AST ≥40 U/l (HR, 1.898; 95% CI, 1.304-2.762, *P* = 0.001), and high *FAM83D* expression (HR, 1.629; 95% CI, 1.138-2.333, *P* = 0.008) were also all independent predictors of the OS (Table 3).

**Table 3:** Cox multivariate proportional hazard model of independent predictors on disease-free and overall survival

Variable	Disease-free survival		Overall survival	
	Hazard ratio (95% CI)	*p* value	Hazard ratio (95% CI)	*p* value
AFP ng/ml (≥100 *vs* < 100)	1.515 (1.063–2.160)	0.021	1.770 (1.128–2.775)	0.013
Tumor size, cm (≥5 *vs* < 5 )	2.768 (1.550–4.943)	0.001	2.622 (1.470–4.678)	0.001
Tumor number (multiple *vs* single)	1.373 (0.953–1.977)	0.089	1.366 (0.949–1.966)	0.093
TNM stage (III-IV *vs* I-II)	1.468 (0.931–2.316)	0.098	1.515 (0.957–2.398)	0.076
PVTT (yes *vs* no)	1.039 (0.682–1.583)	0.860	1.046 (0.687–1.594)	0.833
Metastasis (yes *vs* no)	1.453 (0.924–2.285)	0.106	1.411 (0.893–2.228)	0.140
AST (U/l) (≥40 *vs* < 40)	1.780 (1.226–2.584)	0.002	1.898 (1.304–2.762)	0.001
NLR (≥2.31 *vs* < 2.31)	1.326 (0.938–1.875)	0.110	1.301 (0.920–1.842)	0.137
FAM83D expression (high *vs* low)	1.568 (1.112–2.209)	0.010	1.629 (1.138–2.333)	0.008

CI, confidence interval; AFP, alpha-fetoprotein; TNM, tumor-node-metastasis; PVTT, portal vein tumor thrombus; Metastasis, distant metastasis or lymph node metastasis; AST, aspartate aminotransferase; NLR, neutrophil to lymphocyte ratio.

### Kaplan-meier analysis of DFS in 218 patients with HCC based on statistically significant clinical parameters

**Figure 4:**
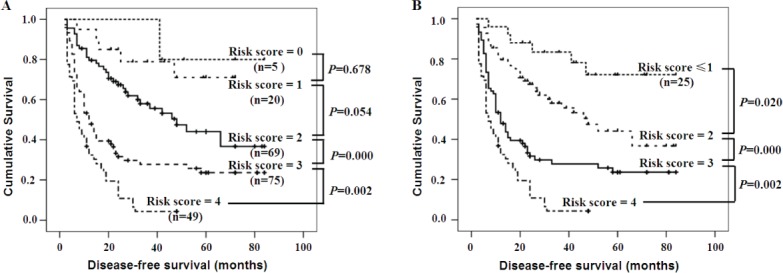
The DFS for patients with various risk scores according to the independent predictors **A.** The Kaplan-Meier curves for the five groups of patients showed that there were no significant differences in the disease-free survival rates of patients with scores of 0 to 2 (all *P* > 0.05), However, there were significant differences in the DFS of patients with scores from 2 to 4 (all *P* < 0.01). **B.** There were also significant differences in the DFS after patients with a risk score = 0 or 1 were merged (all *P* < 0.01).

We established a preoperative prognostic score model by calculating the number of independent predictors (FAM83D, AFP, tumor size, and AST) for each patient. Each factor was allotted a score of 1, and then patients were divided into five categories by their risk scores (RSs) (0, 1, 2, 3, and 4). For example, “RS = 0” means patients without any of the above factors; this group occupied 2.29% (5 of 218). “RS = 4” means patients with all four factors; it occupied 22.47% (49 of 218) of patients carrying all four factors (Figure 4). Because no significant differences were found in the DFS between patients with a risk score = 1 and a risk score = 0 or 2 (Figure 4A, *P* = 0.678, *P* = 0.054, respectively), the groups with a risk score = 0 or 1 were merged to form a score ≤1 group. When using the combination of the four independent predictors, patients with different RSs showed a significant difference in the DFS (RS ≤1 *vs*. RS = 2, *P* = 0.020; RS = 2 *vs*. RS = 3, P = 0.000; RS = 3 *vs*. RS = 4, *P* = 0.002) (Figure 4B). Surprisingly, the proportion of HCC patients with RS = 4 was very high, occupying 22.47% (49 of 218) of total patients, all these patients showed much shorter DFS.

**Figure 5:**
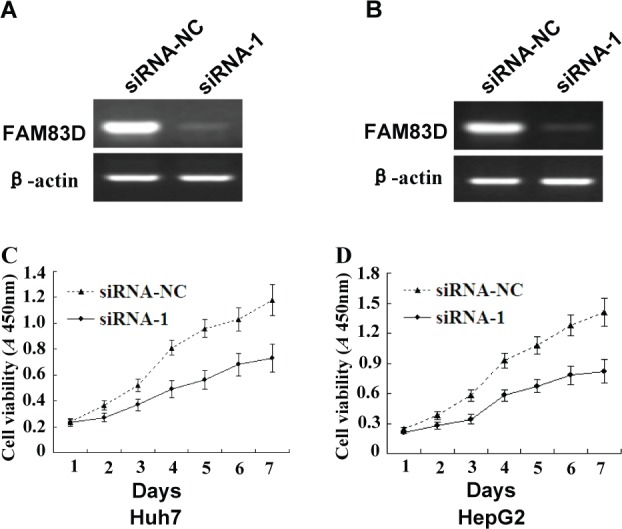
The effects of *FAM83D* silencing on the growth of HCC cells **A.** and **B.** *FAM83D* expression was knocked down in Huh7 **A.** and HepG2 **B.** cells following transient transfection with siRNA-1, which was confirmed by RT-PCR assay. siRNA-NC (as a control) was transiently transfected into Huh7 and HepG2 cells. **C.** and **D.** The growth curves of the Huh7 **C.** and HepG2 **D.** cells with *FAM83D* silencing by siRNA-1 were determined by the CCK-8 assay, and the cells transfected with siRNA-NC served as controls. The experiments were repeated at least three times, and the spots represent the average values of triplicate wells, with standard deviations (SDs) included for each mean value.

### *FAM83D* promotes cell proliferation, migration and invasion, as determined using RNA interference

**Figure 6:**
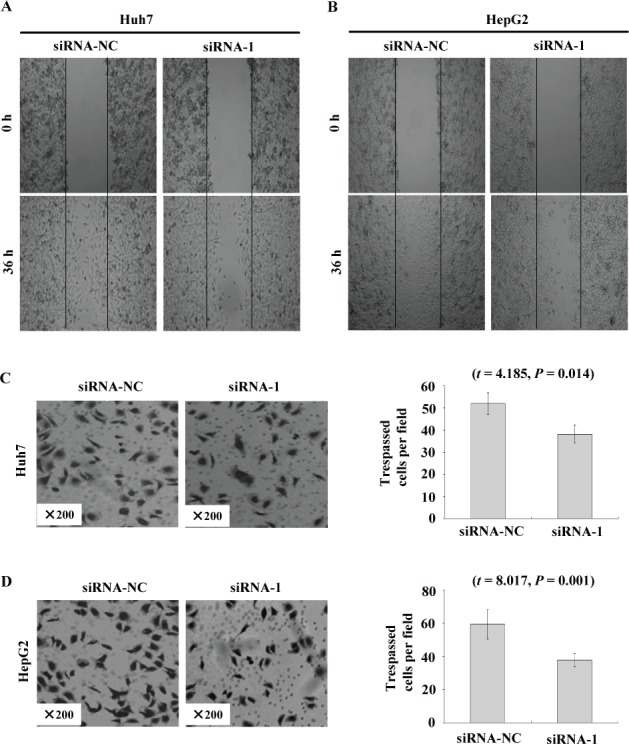
*FAM83D* modulates HCC cell migration and invasion **A.** and **B.** The migration of Huh7 **A.** and HepG2 **B.** cells transfected with siRNA-1 in the wound healing experiments, where siRNA-NC-transfected cells served as a control. **C.** and **D.** The invasion of Huh7 **C.** and HepG2 **D.** cells transfected with siRNA-1 to knock down *FAM83D* expression was evaluated with a Matrigel assay, with siRNA-NC serving as a control. The number of migrated cells is represented by the mean values per field (from at least 5 fields) from 3 independent experiments (right) (mean ± SD).

To further examine the functional role of *FAM83D* in HCC cells, Huh7 and HepG2 cells were transfected with siRNA duplexes against *FAM83D*. Three parallel samples were taken from both the siRNA-NC and siRNA-1 groups. We observed 2 fields (×100) in each sample, for a total of six sections. When the expression of *FAM83D* was silenced in the wound-healing migration assay (the scratch test), microscopic examinations at 0 and 36 h revealed that the migration of the Huh7 and HepG2 cells was inhibited by *FAM83D* silencing (Figure 6A and 6B).

When we inoculated the cells in the upper chambers of a Transwell and counted the Huh7 cells that had penetrated the membrane after 24 hours, 52.00 ± 4.85 and 38.20 ± 4.04 of the cells had penetrated in the siRNA-NC and siRNA-1 groups. The decrease in the number of cells that had penetrated the membrane in the siRNA-1 group compared with the siRNA-NC group was statistically significant (Figure 6C; *P =* 0.014). The numbers of penetrating HepG2 cells in the siRNA-NC and siRNA-1 groups were 59.40 ± 8.71 and 37.80 ± 3.96, respectively. This result demonstrated that there was a significant decrease in cell migration after siRNA-mediated silencing of *FAM83D* compared with the siRNA-NC cells (Figure 6D; *P* = 0.001). After *FAM83D* had been silenced, invasion by both the Huh7 and HepG2 cells was inhibited.

### The DNA methylation status of the *FAM83D* promoter is reduced in HCC tissues

**Figure 7:**
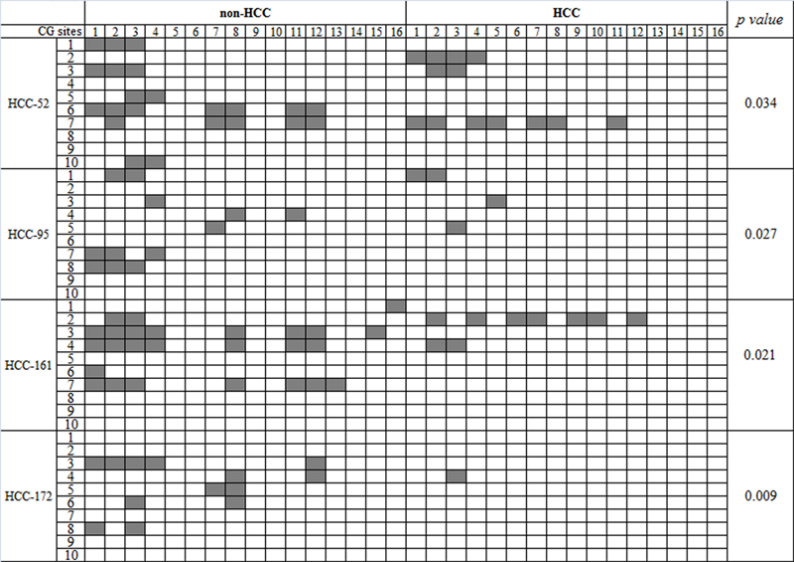
The DNA methylation status of the *FAM83D* promoter in HCC The results from the bisulfite sequencing analysis of *FAM83D* promoter CpG methylation in four pairs of HCC and non-HCC specimens. Each box indicates a CpG dinucleotide within the CpG island in the promoter. Gray box indicates methylation cytosine while while box indicates unmethylation cytosine.

To address whether epigenetic alterations contribute to the dysregulation of *FAM83D* in HCCs, we conducted bisulfite DNA sequencing to characterize the methylation status of the *FAM83D* promoter in four pairs of HCC and non-HCC specimens. The HCC specimens showed *FAM83D* overexpression compared with the expression in the non-cancerous tissues. The sequencing data revealed that the *FAM83D* promoter methylation was significantly reduced in the majority of HCC specimens compared with the paired non-cancerous liver tissues (Figure 7). These findings suggest that the upregulation of *FAM83D* in HCC may be correlated with the methylation levels of the *FAM83D* promoter.

## DISCUSSION

*FAM83D*, the family with sequence similarity 83, member D (also known as C20orf129, CHICA) is located on chromosome 20 in humans [7]. *FAM83D* is involved in mitotic processes to regulate cell division [8]. It may be important for mitotic progression and the equal segregation of chromosomes [9]. *FAM83D* was previously identified as a putative spindle component in a mass-spectrometry-based spindle inventory [10]. It was initially characterized as a novel spindle protein, termed “CHICA”, that was originally identified in a proteomic survey of the human spindle apparatus [10]. Santamaria et al. [8] showed that CHICA coimmunoprecipitates with Kid and is required for the spindle localization of Kid without affecting its chromosome association. Moreover, upon the depletion of either CHICA or Kid (or both proteins simultaneously), the chromosomes collapsed to the poles of monastrol-induced monopolar spindles.

The expression of *FAM83D* appears to be correlated with miR-210 [11]. Recent reports showed that *FAM83D* is involved in breast cancer [12, 13]. However, to our knowledge, there is no information regarding its role in the initiation and/or progression of HCC or the potential clinical implications of *FAM83D* expression in HCC patients. In this study, we confirmed that *FAM83D* was highly expressed in HCC tissues compared with the adjacent liver tissues, and through a statistical analysis, we also found that the *FAM83D* expression level and the level of AFP ( ≥100 ng/ml), clinical TNM stage and presence of PVTT showed a positive correlation (*P* < 0.05). The AFP levels have been widely used for the diagnosis and surveillance of HCC. However, the sensitivity and specificity of the AFP levels for HCC surveillance have some limitations because the levels may be normal in up to 40% of patients with HCC, particularly during the early stages of the disease. Therefore, the identification of other factors affecting the survival of HCC patients, including conventional clinicopathological variables and novel molecular markers, has been a major focus of research in the field. The present study suggests that *FAM83D* has potential as a novel biomarker for the pathogenesis of HCC, and simultaneously detecting AFP and *FAM83D* could improve the prognostic accuracy. The expression level of *FAM83D* was high in both fetal liver tissues and human HCC cell lines, but there was very low or no expression in the normal liver tissues. Therefore, it may be speculated that *FAM83D* plays an important role in maintaining the malignant phenotype of HCC cells, and it might be a gene that is closely related to HCC.

The data obtained from the univariate analysis revealed that high *FAM83D* expression, AFP ≥100 ng/ml, a median size tumor ≥5 cm, multiple tumors, TMN stage III–IV, PVTT, distant metastasis or lymph node metastasis, AST ≥40 U/l and NLR ≥2.31 were all associated with a shorter DFS and OS. In previous studies, the tumor number was shown to be an important determinant of the prognosis of HCC in patients undergoing several types of treatments [14]. Individuals with a single HCC lesion had a relatively higher survival rate than those with multi-nodular tumors. Compared with multiple nodular HCC, a single tumor is strongly suggested to have better prognosis [14]. The main cause of metastatic and recurrent HCC is the invasion of the portal veins by HCC cells, leading to PVTT. PVTT, a unique disseminating form of intrahepatic HCC metastasis, was previously found to be associated with a poor prognosis [15, 16]. It is interesting that the NLR had previously been investigated for it prognostic role in HCC [17–20]. In the present study, we found that an NLR >2.31 was associated with a worse survival for HCC patients, and those with an elevated NLR (>2.3) had a significantly shorter DFS and OS than those with a low NLR (≤2.31).

After adjusting for other confounding factors, high expression of *FAM83D*, AFP ≥100 ng/ml, median tumor size ≥5 cm and AST ≥40U/l were also identified as independent prognostic factors for the DFS and OS by the multivariate analysis in this study. In addition, several studies have previously shown that the level of AFP [21, 22], tumor size [23, 24] and AST [25–27] level were related to the DFS and/or OS in patients with HCC. In addition, a tumor size >5 cm was a significant risk factor for recurrence after liver resection [23, 24], whereas the tumor size and number may indicate whether the HCC is uni- or multi-focal in origin. Relatively small tumors, particularly those <5 cm, are associated with a better prognosis [28, 29], whereas tumors >5 cm are associated with a greater likelihood of vascular invasion and a higher recurrence risk [23, 24, 30]. We used a combination of these 4 independent predictors and separated the HCC patients into four distinct risk score groups with significantly different prognoses. This provided a new risk score for HCC. However, the small sample size of the present study limits its clinical value. It will be necessary to validate the prognostic significance of high *FAM83D* expression in a larger cohort of HCC patients. Finally, personalized therapy and follow-up for patients with early stage HCC can be pursued in the near future based on either the *FAM83D* level alone, or more likely, on the combination of prognostic factors.

The high invasiveness, anti-apoptotic signaling and high invasive potential of the malignant tumor are key factors involved in the development and progression of HCC [31, 32]. Therefore, inhibiting a tumor's proliferation and invasion, while also increasing its level of apoptosis, have been major areas of focus in our research. RNA interference is an effective way to decrease a gene's expression, and it might represent an ideal strategy for cancer therapy [33]. siRNA can inhibit a target gene with specificity and high effectiveness, and siRNAs with high rates of inhibition of the target of interest can be easily detected by screening methods. Our *in vitro* experiments demonstrated that silencing *FAM83D* inhibited the proliferation, migration, and invasiveness of Huh7 and HepG2 cells. These data suggested that *FAM83D* has an essential role in mediating the development and progression of HCC.

In summary, we have demonstrated that *FAM83D* may serve as a powerful prognostic marker and therapeutic target for HCC. *FAM83D* is overexpressed in fetal liver and HCC tissues, and it contributes to the intrahepatic metastasis of HCC (PVTT) and also represents a new independent prognostic factor for HCC. However, the underlying mechanisms by which *FAM83D* exerts its effects require further clarification. Future research should focus on understanding its molecular mechanism(s) of action.

## MATERIALS AND METHODS

### Sources of specimens and cell lines

The 218 HCC tissue specimens used in this research and the adjacent non-HCC tissues were obtained by surgical excision from the livers of HCC patients at the Affiliated Hospitals of Guilin Medical University between November 2001 and April 2007. The diagnosis of HCC was confirmed by clinical and serological features, ultrasonography (US), computed tomography (CT), magnetic resonance imaging (MRI) and pathological examination, and the diagnoses were consistent with the “Primary Liver Cancer Clinical Diagnosis and Staging Criteria”.

The clinicopathological characteristics of these patients, including their age, gender, family history, hepatitis B surface antigen (HBsAg) expression, alpha-fetoprotein (AFP) level, the size and the number of tumors, concomitant liver cirrhosis, history of alcohol consumption, clinical TNM stage, presence of portal vein tumor thrombus (PVTT), development of distant metastasis or lymph node metastasis, postoperative recurrence, aspartate aminotransferase (AST) level and neutrophil-to-lymphocyte ratio (NLR) were collected, and the results are presented in Table 1. In addition, 9 normal tissue samples were taken from the surrounding tissues of patients with hepatic hemangioma, and the identity of the tissue samples was verified pathologically after the operations. The 10 fetal tissue samples were obtained from aborted fetuses in the obstetrics department of the hospital. All samples were frozen in liquid nitrogen and stored at −80°C immediately after the surgical excision, and the study was approved by the Hospital Ethics Committee affiliated of Guilin Medical University. All subjects provided written informed consent based on the Declaration of Helsinki.

Normal liver cell lines (including LO2 and WRL68) and HCC cell lines (including SMMC7721, PLC, MHCC97H, Focus, QGY7701, QGY7703, MHCC97L, Hep3B, MHCC-LM3, MHCC-LM6, Huh7, SK-hep1, YY8103, HepG2, BEL7402, BEL7404 and BEL7405) were cultured in our laboratory.

We investigated HCC patients with long-term follow-up after surgery. The follow-up studies included serum AFP and US every 2 months and chest radiography every 6 months during the first two postoperative years and at 3-6-month intervals thereafter. CT or MRI was performed if recurrence was suspected due to an abnormal AFP test or US examination. The mean postoperative follow-up term was 35.5 months (median, 21.0 months; range, 2.0 to 84.0 months). The disease-free survival (DFS) was measured from the date of surgery to the date of recurrence, metastasis, death or the last follow-up. The overall survival (OS) was measured from the date of surgery to the date of death or the last follow-up.

### RNA extraction and cDNA synthesis

Total RNA was isolated from HCC cell lines or frozen tissue samples that were pulverized under liquid nitrogen and extracted using the TRIzol (Invitrogen, Carlsbad, CA, USA) reagent. To reduce the risk of genomic DNA contamination, DNase treatment was performed on 1 to 2 μg of RNA by adding 2 U of DNase I (Invitrogen), 1 μl DNase buffer, and 0.4 μl RNase Out for 15 min at room temperature. The concentration of RNA was determined by spectrophotometry, and the total RNA integrity was monitored by visualization of ribosomal RNAs (28S and 18S) on 1.2% agarose gels. First-strand cDNA was synthesized using a PrimeScript RT Reagent Kit (TaKaRa) according to the manufacturer's instructions.

### Determination of gene expression by reverse transcription–PCR and quantitative real-time PCR

Based on the GenBank cDNA sequences, we designed primer sequences for *FAM83D* (NM_030919.2) and *ACTB* (encoding β-actin) using the Primer Premier 5.0 program. In a PubMed BLAST comparative analysis, the designed primer sequences showed comparatively good specificity (the primers were synthesized by Shanghai Biological Engineering Co., Ltd). For the reverse transcription-polymerase chain reaction (RT-PCR), the upstream primer sequence for *FAM83D* was 5′- AACCACTGACTTC CACAATCCT-3′, and the downstream primer sequence was 5′-CAAAACAAACCC CTGTATCCAT-3′, which resulted in an amplified fragment of 496 bp. *ACTB* was used an internal control with an upstream primer sequence of 5′-TCAC2CCACACTGTGCCC ATCTACGA-3′ and a downstream primer sequence of 5′-CAGCGGAACCGCTCA TTGCCAATGG-3′ to amplify a 295-bp fragment. The PCR reaction was performed using the TaKaRa PCR kit using a 20-μl reaction volume. The reaction conditions were 94°C for 5 min, followed by 35 cycles (for FAM83D) or 25 cycles (for *ACTB*) of 94°C for 30 s, 55°C for 30 s, 72°C for 30 s; then 5 min at 70°C, followed by storage at 4°C. The PCR products were run on 2% agarose gels and visualized after staining with ethidium bromide.

The qRT-PCR was performed according to the instructions for the SYBR Premix Ex Taq. We designed the primer sequences to amplify *FAM83D* as follows: 5′-ATGGACGGATGGCAAATTAAAC-3′ (sense) and 5′-CTGCTCTGGAAGT GAGACAGGA-3′ (antisense), with the length of the amplified fragment being 141 bp. The primer sequences for the internal control (*ACTB*) were 5′-GACAGGATGCAGAAGGAGATTACT-3′ (sense) and 5′-TGATCCACATCTGCT GGAAGGT-3′ (antisense) (with the length of the amplified fragments being 142 bp).

The qRT-PCR amplification and data analysis were performed using the ABI Prism 7500 Sequence Detector System from Applied Biosystems (Foster City, CA, USA). Each cDNA sample was mixed with 15 μl of master mix (SYBR^®^ Green PCR Master Mix, Applied Biosystems). For PCR, an identical amplification protocol was used, and consisted of an initial denaturation step at 95°C for 10 min, followed by 40 cycles (denaturation at 95°C for 2 sec, annealing at 55°C for 5 sec, extension at 72°C for 15 sec) and fluorescence acquisition at 72°C. The relative *FAM83D* mRNA expression was calculated as described in our previous report [5].

### Immunohistochemistry (IHC) assay

Tissue microarrays were prepared according to the following method. Formalin-fixed, paraffin-embedded tissue blocks and the corresponding hematoxylin-eosin (H&E)-stained slides were overlaid for tissue microarray sampling. The HCC and non-HCC tissue array slides with normal tissue controls (12 cases/24 cores) were reviewed by two histopathologists, and representative tumor areas free from necrotic and hemorrhagic materials were premarked in the paraffin blocks.

The tissue microarray slides were deparaffinized in xylene, rehydrated through a graded series of ethanol, subjected to antigen retrieval by pressure cooking for 3 minutes in ethylenediamine tetraacetic acid (EDTA) buffer (pH = 8.0). Then, the slides were washed in phosphate-buffered saline (PBS) and immersed in 3% hydrogen peroxide for 20 minutes to block the endogenous peroxidase activity. The slides were preincubated with 10% normal goat serum at room temperature for 30 minutes to reduce nonspecific binding. Subsequently, the slides were incubated with rabbit polyclonal anti-FAM83D (lot^#^: SA110329BV, ABGENT Company, 1:200 dilution) antibodies overnight in a moist chamber at 4°C, washed in PBS, and incubated with a biotinylated goat anti-rabbit antibody for 1 hour at room temperature. The samples were then stained with 3, 3- diaminobenzidine tetrahydrochloride (DAB). Finally, the sections were counterstained with Mayer's hematoxylin, dehydrated, and mounted. A negative control was obtained by replacing the primary antibody with normal rabbit or mouse IgG. Semi-quantitative IHC detection was performed to determine the FAM83D protein levels, and the stained tissue sections were assessed using a 4-point scale as follows: positive cell counts, grades 0-3 (0, no positive cells; 1, <25% positive cells; 2, 25%-50% positive cells; 3, >50% positive cells). The slides were separately reviewed under light microscopy by two pathologists.

### The design and synthesis of RNAi fragments

Three siRNAs against *FAM83D* were designed using the Whitehead Institute Web Server (http://jura.wi.mit.edu/bioc/siRNAext/) and were chemically synthesized (Shanghai GenePharma Co.). The sequences of the siRNAs targeting different coding regions of the gene were as follows: the sequence of the siRNA-1 sense strand was 5′-GCAGUAACUUGGUAAUUCUTT-3′, and the sequence of the antisense strand was 5′-AGAAUUACCAAGUUACUGCTT-3′; the sequence of the siRNA-2 sense strand was 5′-CGGACUAUCACAGGAAAUATT-3′, and the sequence of the antisense strand was 5′-UAUUUCCUGUGAUAGUCCGTT-3′; the sequences of the siRNA-3 sense strand was 5′-GCAGUCUCAUAAGAUUAUATT-3′, and the sequence of the antisense strand was 5′-UAUAAUCUUA UGAGACUGCTT-3′. The sequences used for the negative control (siRNA-NC) were 5′-CGUCAGAGUAUACUAAUAUTT-3′ (sense) and 5′-AUAUUAGUAUACUCUGACGTT-3′ (antisense). The RNAi fragments were synthesized by Shanghai GenePharma Co., Ltd.

### Cell culture and transient transfection

The HCC cell lines Huh7 and HepG2 were cultured in Dulbecco's modified Eagle medium (DMEM) with high glucose (Gibco), and complete culture solution was used for all other cell lines, which was supplemented with 10% fetal bovine serum (FBS; Gibco), 100 g/l penicillin, and 100 g/l streptomycin (Invitrogen). All cells were cultured at 37°C, and the CO_2_ concentration was 50 ml/l. The cell growth was observed every 2 days, and 2.5 g/l trypsin digestion was conducted for subculturing. The cells were transferred to 6-well plates, and serum-free medium was used when the cells reached approximately 70% confluence. We used Lipofectamine^™^ 3000 (Invitrogen) for the transient transfection of cells with siRNA-1, siRNA-2 and siRNA-3. A negative control group (siRNA-NC) and a blank group were included as controls. After 4 to 6 hours of transfection, the cells were switched to DMEM complete medium, and the cells were collected after 72 hours of culture. The TRIzol reagent was used for total RNA extraction and the analysis of the inhibitory effects of the RNAi.

### CCK-8 assay for cell growth

We selected the siRNA sequences (siRNA-1) with the best inhibitory effects to conduct the subsequent experiments. After 24 hours of cell transfection, we adjusted the cell concentration of each group to 2×10^4^/ml, inoculated the cells onto 96-well plates with 100 ml of medium per well, and placed them in humidified incubators at 37°C and 5% CO_2_ for cultivation. The cells were grown to 30%-50% confluence and then transfected with the synthetic siRNAs at final concentrations of 50 nM using the Lipofectamine^™^ 3000 transfection reagent, according to the manufacturer's instructions. The cells were then sub-cultured at 24-hour intervals for 7 days. The cell viability was measured using CCK-8 according to the manufacturer's instructions. In brief, 10 μl of CCK-8 solution was added to each well of the plate, and the plate was incubated at 37°C for 1 hour. The absorbance was measured at 450 nm to assess the cell viability. All experiments were independently repeated at least 3 times.

### The scratch test to determine the cell migration

Cells in the logarithmic growth phase were plated into 6-well plates at 37°C, 5% CO_2_/95% air for 24 hours. We used the lance point of a 2-μl pipette to make a scratch in each well, and we applied serum-free media to gently clear the cells from the scratched area. For each group, 3 parallel samples were evaluated for the ability of the cells to grow into the scratch.

### Test of the *in vitro* cell invasion

A total of 600 μl of DMEM or MEM complete medium was put into the lower chamber of a Transwell plate (including 100 g/l penicillin, 100 g/l streptomycin and 10% FBS). The upper chamber was filled with 100 μl DMEM, and the plates were allowed to equilibrate overnight in an incubator. The cultured cells were detached from the wells and then resuspended in DMEM. The media was removed from the Transwell plates, and the upper chamber was washed 3 times with DMEM and returned to the lower chamber. The upper chamber was filled with 100 μl of cell suspension, with 1 × 10^4^ cells per well, and the lower chamber was filled with DMEM medium containing 20% FBS. After 24 hours at 37°C, the upper chamber was removed and cleaned using cotton balls that had been dipped into PBS solution. Then, 70% carbinol was used to fix the cells on the membrane for 20 minutes, followed by staining with 0.5% crystal violet (CV) for 5 minutes and 3 washes with water. Finally, the membrane was placed on a glass slide, with its bottom side facing up. The membrane was then sealed on the slide with a cover after dripping resin onto it. Two double-blind experimenters counted 5 cells at random in their field of vision 100 times, and the average value was considered to represent the numbers of cells that had invaded across the Transwell membrane in the siRNA-1 and siRNA-NC groups. Each group included 3 parallel samples.

### Bisulfite DNA sequencing

Genomic DNA was extracted from the tissue samples using the DNeasy Tissue Kit (Qiagen) according to the manufacturer's instructions. Bisulfite treatment of the genomic DNA was performed using the EpiTect Bisulfite Kit (Qiagen) according to the manufacturer's instructions. For bisulfite DNA sequencing, the CpG island was amplified using 5′- TTAAAGTATTGAGAGTTTAGGAGTAG-3′ (forward) and 5′- CATTAACTACTTTCCTTCCAATAAC-3′ (reverse), which are primers specific for the *FAM83D* gene promoter. The PCR products were purified and subcloned into the pMD18-T vector (TaKaRa). Colonies were selected at random colonies for sequencing.

### Statistical analysis

The SPSS13.0 software program was used for the statistical analyses. The Pearson Chi-square test was used to compare qualitative variables, and quantitative variables were analyzed by the independent *t-*test. The survival probability was estimated by the Kaplan-Meier method, and the log-rank test was used to compare the survival curves between the groups. Stepwise Cox proportional hazard models were used to identify independent predictors associated with the DFS and OS. Differences between the mean values were considered significant when *P* < 0.05.

## SUPPLEMENTARY MATERIAL FIGURE


