# Supplementary material for: Upregulation of FAM83D affects the proliferation and invasion of hepatocellular carcinoma
Source: Oncotarget. 2015 Jun 10;6(27):24132–47. doi: 10.18632/oncotarget.4432 (PMC4695175; doi:10.18632/oncotarget.4432)
Supplement: Supplementary file 1 [file oncotarget-06-24132-s001.pdf]

# Upregulation of *FAM83D* affects the proliferation and invasion of hepatocellular carcinoma

**Supplementary Material**

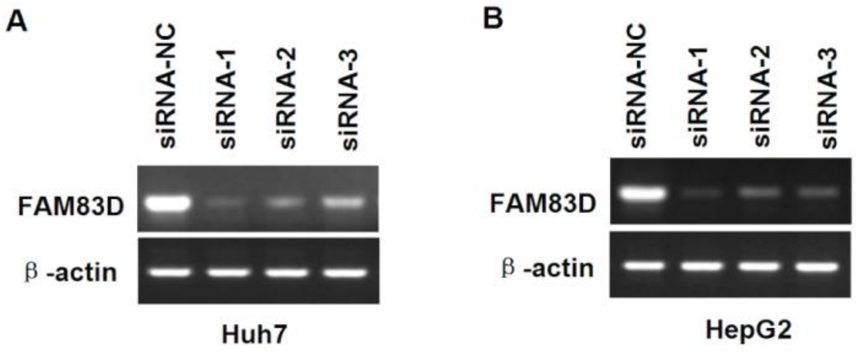


**Figure S1: The results of a screen for the most efficient RNAi fragment for the knockdown of *FAM83D*.** (A and B) siRNA-1, siRNA-2 and siRNA-3 were used to knock down *FAM83D* in Huh7 (A) and HepG2 (B) cells, as demonstrated by RT-PCR. siRNA-NC was used as a control.
